# Carbonate of Creasote in Pneumonia

**Published:** 1901-11-23

**Authors:** 


### CARBONATE OF CREASOTE IN PNEUMONIA.

It is perhaps a bold thing to advocate any particular drug as having a curative action in a disease like pneumonia, so strongly has professional teaching of late years held that the patient should be supported and the complications if possible controlled, but that so far as the disease itself is concerned, all we can do is to stand aside, unless indeed we are able by aid of an antitoxic serum to put a limit to the morbid process. Nor is faith in the suggestion increased when one finds that the drug recommended is a form of creasote, for has it not been abundantly shown that patients may be soaked with antiseptics without any effect being produced upon the microbes by which their tissues have been invaded? But in advocating the use of carbonate of creasote, Dr. Leonard Weber[1] of New York puts forward a suggestion which we must not cast aside as entirely inappropriate, little as it may be capable of proof—namely, that although the drug may be neither antipyretic nor bactericidal, it may be antidotal to the pneumonia toxins. It is by no means improbable that if ever the drug treatment of acute diseases should reach a scientific basis, it will be by the discovery of chemical antidotes for bacterial toxins—antitoxic drugs to take the place of antitoxic sera. Perhaps we already have such drugs without knowing it. Rheumatism is said to be a bacterial disease, yet no one can believe that its microbes are killed by alkalies and salicylates. We cannot deny, however, that these drugs have an influence upon the symptoms. Do they then act by neutralising the rheumatism toxin? And so with pneumonia we may despair perhaps of finding a drug which will kill microbes without killing at the same time the man in whom they breed. But it by no means follows that we need despair of discovering a chemical product which shall neutralise their toxic products, and it is by these that much of the danger of the malady is caused. At any rate, there the suggestion is! After giving details of a number of cases in which he had used carbonate of creosote, Dr. Weber states that this drug exercises a remarkably beneficial and uniform influence upon the pneumonic patient. He says that according to present working theories the illness of the patient suffering from pneumonia is caused by specific toxins formed by certain pathogenic germs, and his recovery is dependent upon the speedy production of serum antitoxins, which must be sufficient in quantity and quality to overcome the toxins circulating in the blood. In the majority of the cases the system is equal to the demand; but in a considerable number it is not, and the patient succumbs. When a typical case of pneumonia ends by crisis, the patient rests quietly, breathing easily, and the temperature is normal or subnormal. But the affected lung is found to be still in the same state of hepatisation as the day before. Very much the same thing occurs, he says, after the administration of creasotal. This is shown in the cases cited, and in many reported by other observers. About 1 drachm of the drug having been taken the temperature goes down to normal, and the patient feels much better generally. If the drug be now discontinued the temperature will go up again: we resume it and continue at the rate of 50 or 60 grains per day, and then the temperature will go down and stay down and the affected lung will begin to clear up. The drug was given in gelatine capsules made after the following formula:—℞ Carbonate of creasote, ½ oz.; sapon. medicat., 1 dr.; M. F. capsulæ 60. Two to be taken every three hours.

[1] New York Medical Record, Nov. 2.

